# Supplementary material for: Are carbon emissions trading and green financial instruments synergistic? -Comprehensive quantitative research based on content analysis
Source: PLoS One. 2024 Mar 7;19(3):e0298601. doi: 10.1371/journal.pone.0298601 (PMC10919601; doi:10.1371/journal.pone.0298601)
Supplement: S1 Data — (ZIP) [file pone.0298601.s001.zip › Data/地级市Z_F工作报告.zip!/║╙▒▒/╨╧╠¿╩╨/└┤╫╘╣1⁄2╓┌║┼í╛┬φ┐╦╩2╛▌═°í┐_2012.docx]

各位代表：

　　现在，我代表市人民政府，向大会作工作报告，请予审议，并请各位政协委员和列席会议的同志提出意见。

　　一、2011年工作回顾

　　过去的一年，是“十二五”开局之年，也是外部环境异常复杂的一年。面对严峻形势和繁重任务，我们在省委、省政府和中共邢台市委的坚强领导下，坚持以科学发展为主题，以加快转变经济发展方式为主线，狠抓重点求突破，深入基层解难题，全力以赴“增投入、扩总量，调结构、促升级，惠民生、保稳定”，有效应对了经济下行压力加大、农业严重干旱等挑战和困难，较好地完成了市十三届人大四次会议确定的目标任务，实现了“十二五”开门红。

　　（一）经济增长总体较快。全市生产总值完成1426.3亿元，增长11.6%。全部财政收入151.4亿元，增长13.9%；地方一般预算收入70.6亿元，增长23.6%。地方一般预算收入占全部财政收入的比重达到46.6%，提高3.7个百分点。所有县（市）全部财政收入均超亿元，实现了历史性突破。财政支出213.1亿元，连续四年超过财政收入，再创历史新高。农业效益稳步提升，粮食总产达464.8万吨，实现“八连增”，棉花总产、单产均创历史新高，成功举办首届中国核桃节，新增年销售收入5000万元以上农业产业化龙头企业9家、省部级畜禽标准化规模养殖示范场49家、设施蔬菜6.2万亩。工业经济实力提升，规模以上工业增加值完成568.7亿元、增长15.7%，实现利税185亿元、增长19%。服务业活力增强，天一广场商业综合体竣工开业，与沃尔玛、乐购、红星美凯龙等签订了合作协议，全国高速公路出行信息服务中心落户邢台，我市被列为全省唯一一家全国家政服务体系建设试点市；好望角等物流园区加快提升；新增国家4A级景区4家；新增村镇银行3家，小额贷款公司11家，数量均居全省首位。

　　（二）项目投资势头活跃。全社会固定资产投资完成1048.9亿元，增长25.5%。列入省重点项目100项，居全省第四位，完成投资300亿元；安排市重点项目217项，完成投资340亿元，均超额完成年度投资计划。今麦郎提质扩能等56个项目投产达效，中航上大特种合金材料等121个项目开工建设，宁晋盐矿开发等5个重大前期项目提前开工。加大了瓶颈破解力度，积极协调调度，保障了用电等要素供应；全市金融机构贷款余额达到946.9亿元，增长16.3%；为中小微型企业新增担保贷款5.5亿元。

　　（三）结构调整成效明显。装备制造、新能源、煤盐化工、新型建材等主导产业不断壮大，金后盾汽车零配件及方舱生产线项目加快建设，晶龙400兆瓦太阳能电池、旭阳30万吨焦油等项目竣工投产，沙河玻璃制造及深加工国家新型工业化示范基地通过工信部认定；3家企业跨入中国500强，其中冀中能源入围世界500强。钢铁、纺织服装等传统产业和自行车、轴承等特色产业改造提升步伐加快，169个技改项目列入省重点，争取中央、省技改专项资金2.2亿元，居全省第一位。新能源汽车、新材料等新兴产业加速扩张。园区创建实现突破，新增省级开发区、工业聚集区17家，居全省第二位。创新能力进一步提升，新增国家级企业技术中心1家、省级工程技术研究中心3家、中科院专家工作站1家，晶龙集团被确定为国家级技术创新示范企业，3家企业被认定为国家火炬计划重点高新技术企业；新增中国驰名商标7件，居全省第一位。节能减排扎实推进，我市被授予全省唯一一家“十一五”节能减排先进市，万元生产总值能耗、化学需氧量、二氧化硫等完成省定目标。

　　（四）城乡统筹稳步推进。调整了“一城五星”部分行政区划,完善了财政体制，对“五星”及各区实行统筹发展的激励性政策，市区连接“五星”绿色长廊和“五星”之间快速路加快建设，“五星”出租车与市区实现同城化管理。邢汾高速、邢黄高速、京港澳高速改扩建、邢黄铁路等重大交通项目开工建设，环城高速加快形成，邢和铁路、邢台机场民航复航前期工作进展顺利；南水北调、西线引黄等工程加快推进。市区城镇化建设百项重点工程启动实施，国家级园林城市创建工作全面铺开，邢台新区起步区、七里河新区加快建设，城市原点顺利落成，城中村、旧城区、危旧房、棚户区改造步伐加快，桥西区断头路全部打通，桥东区启动11条、打通3条，市区水系和水生态修复工程启动实施，市区防洪标准达到百年一遇，市图书馆即将向社会开放，市广电中心、邢台学院体育馆主体完工，万峰五星级酒店、百泉湾温泉度假酒店竣工开业，辰光五星级酒店开工建设，晶龙五星级酒店具备开工条件。对市区出入口、16条主要街道、噪声污染等进行了集中整治，大气污染综合整治专项行动取得阶段性成效，白马河垃圾场封场、矸石山搬迁进展顺利；邢台电缆厂等6家企业退市进园工作扎实推进。市区新增集中供热面积211万平方米、民用燃气用户2.6万户，新建便民菜市场34个。各县（市、区）实施了一批重点工程，功能进一步完善，新增宁晋、巨鹿、南和为省级园林县城；319个省级新民居示范村加快建设，开展农村环境卫生集中整治，农村面貌明显改观。

　　（五）改革开放继续深化。重点领域改革进展顺利，组建河北顺德投资集团，融资15.6亿元；整合职教资源，市职教中心、经贸学校并入邢台技师学院，市农业学校、财贸学校、工业学校三校合并成立了邢台现代职业学校；顺利完成医药卫生体制改革阶段性任务，政府办基层医疗卫生机构基本药物零差率销售全面实行，公立医院改革试点扎实推进。开放招商成效明显，回购长征汽车厂全部股权得到省政府批准，与中航集团合作的重型车基地项目取得实质性进展，中建材、燕京啤酒等进入我市开展合作或并购，引进艾德LED、好孩子北方工业园等一批优质项目；邢台海关大楼主体完工，与德国施-迈县建立了友好城市关系，与国家开发银行、中国电力投资集团、河北科技大学签署了合作协议，新增上市企业1家。实际利用外资3.62亿美元、增长41.6%，引进国内市外资金230亿元、增长42.8%；实现进出口总额21.8亿美元、增长19.8%，其中出口11.6亿美元、增长20.6%。

　　（六）人民生活持续改善。城镇居民人均可支配收入16592元、增长12.5%，农民人均纯收入5814元、增长17.1%。全市用于民生方面的财政支出达157亿元，连续六年保持增长，占总支出比重超过七成。在全省率先制定实施市区困难家庭综合救助办法，不让困难家庭因物价上涨而影响基本生活，家庭成员有劳动能力而不能就业，因费用负担而不能就医、就学、住房、用水、取暖、维权、创业，发放救助资金和减免各项费用735.2万元，救助市区困难家庭12615户；率先大规模引进高层次人才，面向“211”名校和国内外高校，公开招录（聘）高层次人才780名，其中市直520名，并建立了人才后备库；率先推行“72小时就业服务”，城镇新增就业5.78万人，登记失业率3.9%，农村劳动力向非农产业转移18.6万人（次）。社会保险扩面顺利推进，失业保险实现市级统筹，城镇居民和新型农村社会养老保险试点县（市）达到11个，新农合参合率达到96.1%；提高了公务员津贴补贴、企业职工最低工资、退休人员基本养老金和城乡低保标准。市区临时环卫工人工资由620元/月提高到1040元/月。超额完成省下达保障房建设任务，开工建设保障性安居工程住房27421套，改造农村危房12198户。扶贫开发力度加大，争取国家和省新一轮扶持重点村387个，数量居全省第一，2.5万贫困人口实现稳定脱贫。解决了41万农村人口饮水不安全问题。

　　（七）社会事业全面进步。教育事业快速发展，新建、改扩建市区小学8所，开工中小学校舍安全工程项目693个，中小学教育教学质量显著提升。改善医疗卫生条件，市人民医院扩建工程启动拆迁，市眼科医院迁建工程开工建设，基层医疗卫生服务体系不断健全，新建标准化村卫生室117个，市区社区卫生服务中心覆盖率98%以上。群众文化生活日益丰富，成功举办纪念郭守敬诞辰780周年暨太行山文化节等活动。加强人口计生工作，低生育水平保持稳定。全民健身活动广泛开展，成功举办市第十三届运动会。平安邢台创建取得新成效，建立了社会面“大巡控”机制，“两抢一盗”发案率大幅下降，打掉涉恶团伙7个。着力加强和改进群众工作，信访稳定取得积极成效。普法宣传深入开展，社会管理创新不断加强，安全生产和食品药品安全形势保持稳定。应急机制进一步完善，有效应对有气象记录以来12小时内降雪量最大的暴雪天气，保证了企业生产和社会生活正常秩序。对口援建新疆若羌县的河北“双语”幼儿园在全省率先交付使用。双拥共建、民族宗教、外事侨务、防震减灾、人防气象、老龄妇幼、统计、档案、残疾人、地方志等事业取得新成绩。

　　（八）政府建设不断加强。深入开展创先争优和“双提”活动，将一批新投产省重点项目和商贸流通龙头增列为市长联系企业，公开曝光处理损害发展环境典型案件12起，发展环境进一步优化。在全省率先建立行政权力长效动态管理机制，大力规范行政职权和自由裁量权，废止、修改规范性文件36件，依法行政水平得到提升。积极推进行政权力公开透明运行，廉政建设和反腐败斗争取得新成效。市行政服务中心建设全面加强，审批服务事项按时办结率达100%。建筑劳务实名制一卡通网络管理系统在全市推行。高度重视向人大常委会报告工作，自觉接受人大法律监督、政协民主监督和社会监督，办理市人大代表建议274件、市政协提案603件，按时办结率均为100%。

　　各位代表，过去的一年，挑战非同寻常，成绩来之不易，这是省委、省政府和市委正确领导的结果，是全市人民众志成城、共同努力的结果。在此，我代表市人民政府，向全市人民，向人大代表、政协委员，向各民主党派、工商联、无党派人士和人民团体，向各位老领导、老同志，向驻邢人民解放军、武警官兵和政法干警，向中省直驻邢各单位，向关心和支持邢台发展的海内外朋友，致以崇高的敬意和衷心的感谢！

　　同时，我们也清醒地看到，尽管去年绝大多数目标任务完成或超额完成，但仍有服务业增加值、城镇化率、氮氧化物排放量等3项主要指标未完成预期目标。经济社会发展中还存在一些突出问题：

　　一是经济总量小、增速不快、结构不优、效益不高的矛盾还未根本解决。人均生产总值、财政收入等指标仍居全省后列。农业基础设施薄弱，龙头企业少，产业化水平不高；工业企业技术装备差，资源、能源依赖度高；服务业占比较低。产品科技含量低、附加值低，初级产品、原材料产品占比高。

　　二是部分企业尤其是中小企业发展面临困难。原燃料价格居高不下，资金、劳动力等要素制约凸显，生产成本增加，经营出现困难。

　　三是县域经济规模小、实力弱、发展水平低。全省“三十弱”的县当中我市占8个，“三十强”中我市仅有1个。

　　四是保障和改善民生压力较大。由于物价涨幅较大，低收入群体和困难家庭生活受到影响，社会事业欠账依然较多。

　　五是发展环境仍需优化。少数政府部门和工作人员思想还不够解放，事业心、责任心淡薄，缺乏担当和创新，抓工作漫不经心、跑升迁绝不松心；作风漂浮，执行力不强，反映情况打埋伏，汇报工作报虚功，不推不动，甚至推也不动；推诿扯皮、吃拿卡要、消极腐败、官僚主义等现象仍然存在。对于这些问题，我们将采取更加有力的措施，下大力切实加以解决。

　　各位代表，我们一定倍加珍惜取得的成绩，始终保持清醒头脑，坚定信心，振奋精神，更加兢兢业业、扎扎实实地做好各项工作，决不辜负全市人民的殷切希望!

　　二、2012年工作的总体要求和主要目标

　　今年是全面落实省、市第八次党代会精神的第一年，是实施“十二五”规划承上启下的关键一年，我们将迎来党的十八大胜利召开，做好各项工作，意义重大。

　　展望全年，形势依然复杂严峻。挑战不容忽视。世界经济复苏乏力，不确定、不稳定因素较多；国内经济增速趋缓，通胀压力仍然较大，能源、环境、土地、产业准入等政策约束进一步强化，节能减排压力越来越大，同时中央继续实施积极的财政政策和稳健的货币政策，对我们落实配套资金、增加民生支出和优化投资结构、提高项目质量提出了更高的要求；各兄弟市竞相发展，比环境、抢项目、争资金，竞争日趋激烈；我市面临经济总量不足、质量不高的双重制约，跨越发展、和谐稳定的双重任务，既要爬坡、又要赶路的双重压力，不进则退、慢进也退。机遇更加难得。全球产业重组和转移步伐加快，科技创新成果不断涌现；中央确定了“稳中求进”的经济工作总基调，“南资北移、东企西进”步伐加快；冀中南地区上升为国家层面的重点开发区域，省加大对黑龙港流域和太行山区的扶持力度；我市“东出西联、南承北接”交通项目加快建设，东西阻隔局面即将彻底打破，开发区、工业聚集区等各类园区的承载能力增强，一大批产业支撑项目开工建设或投产达效，前进的基础更加坚实。我市政治安定、社会稳定、人心思进，特别是市第八次党代会明确提出了在科学发展道路上奋力走向振兴的奋斗目标，全市上下思想统一、精神振奋，形成了加快发展、摆脱落后的浓厚氛围。

　　总之，面对复杂多变的发展形势，面对稍纵即逝的难得机遇，我们必须进一步增强责任意识、机遇意识、忧患意识，坚持发展第一要务不动摇，保持较快发展速度不松懈，开拓创新，奋力拼搏，努力开创经济社会发展的新局面。

　　今年政府工作的总体要求是：高举中国特色社会主义伟大旗帜，以邓小平理论和“三个代表”重要思想为指导，深入贯彻落实科学发展观，全面落实省、市第八次党代会和中央、省、市经济工作会议精神，以科学发展为主题，以加快转变经济发展方式为主线，紧紧围绕转型升级、赶先进位两大任务，着力深化改革开放和创新，着力推进结构调整和节能减排，着力建设优质新项目、培植可持续增长点，着力保障改善民生和促进社会和谐，着力提高城乡统筹发展水平，致力还邢台青山绿水、坚决走生态发展之路，努力实现更好更快更大发展，奋力开启建设经济强市、和谐邢台的新征程，在走向振兴的道路上迈出坚实步伐。

　　经济社会发展的主要预期目标是：生产总值增长11%以上。全部财政收入增长14.5%，地方一般预算收入增长15.5%。全社会固定资产投资增长20%，规模以上工业增加值增长14%以上，社会消费品零售总额增长17%，出口总值增长10%，实际利用外资增长10%。万元生产总值能耗、主要污染物排放量降低率完成省下达任务。城镇居民人均可支配收入、农民人均纯收入均增长9%以上。城镇登记失业率控制在4.2%以内，居民消费价格涨幅控制在4%左右，人口自然增长率控制在8.85％。以内。

　　实现今年经济社会发展目标，必须坚定不移地把“增投入、扩总量，调结构、促升级，惠民生、保稳定”作为着力点和主攻方向，坚持以项目为支撑、以招商求突破、以环境作保障，努力扩张经济规模；坚持一产抓特色、二产抓提升、三产抓拓展，促进三次产业协调发展；坚持创新发展、集约发展、绿色发展，加快转变经济发展方式；坚持做强中心城市、壮大县域经济、建设社会主义新农村，提升城乡统筹发展水平；坚持尽心竭力保障和改善民生、繁荣发展社会事业，大幅度提高人民群众的幸福指数。

　　各位代表，发展的目标激励着我们，人民的期待鞭策着我们。只要我们坚持以只争朝夕的精神抓工作，以事争一流的激情干事业，全力以赴推进科学发展、创新发展、生态发展，就一定能够实现今年的各项目标，向全市人民交上一份满意的答卷!

　　三、奋发有为地做好经济工作

　　牢牢把握转型升级、赶先进位的关键环节，突出重点，突破难点，打造亮点，着力做好八个方面的工作：

　　（一）以项目建设为支撑，保持经济平稳较快增长。发挥投资第一拉动力作用，努力在项目建设规模和质量上实现新提升。

　　一是狠抓重点项目建设。抓住与中航合作的难得机遇，集中力量，强力推进，力促中航集团邢台重型车基地项目尽早开工建设，带动我市汽车产业振兴发展，早日实现邢台人民再现汽车产业辉煌的强烈愿望。积极推动与新兴际华集团的战略合作，促进投资超百亿元的产业园区项目尽快落地开工；加快推进宁晋盐化工园区、深港电子信息产业基地、好孩子童车北方工业园等重点项目建设，力争早日投产达效。

　　二是加强项目谋划储备。顺应国家产业政策导向和市场需求，以培育壮大四大千亿产业、新兴产业和现代服务业为重点，积极谋划筛选一批优质大项目、好项目，充实项目储备库，做到让项目等机遇，决不让机遇等项目。

　　三是进一步优化项目结构。严把项目建设能评、环评关口，坚决杜绝高耗能、高污染、低效益项目上马。积极与大专院校开展对接合作，进一步提升科技含量高、效益好项目的投资比重。提高项目质量，提升项目投资强度、容积率、回报率，力争一批项目列入省重点盘子。

　　四是努力破解项目建设瓶颈。加强项目策划包装，加大争跑力度，争取上级政策、土地、资金支持。抓好土地挖潜，盘活存量土地，规范项目用地管理，促进土地集约、高效利用。拓宽项目融资渠道，积极帮助项目单位通过银行贷款、票据等增加融资，鼓励和引导民间资本参与项目建设。

　　总之，项目建设事关全局、事关未来。我们一定要咬定青山不放松，一步一个脚印地把项目建设抓下去，为全市经济快速发展提供有力的支撑！

　　（二）以发展现代农业为重点，扎实做好“三农”工作。以工业化的理念抓农业，发挥优势、整合要素、打造特色、提升整体竞争力，促进农业增产、农民增收、农村繁荣，努力建设社会主义新农村。

　　一是依靠科技提高粮食产量。加强农技推广体系建设，进一步加大“两晚”等实用技术和优良品种、机耕机作的推广力度，抓好粮食生产核心区和部级万亩高产创建示范片建设，力争建成5个“吨粮县”。

　　二是优化农业产业结构。巩固提高81个省部级、创建30个市级畜禽标准化规模养殖示范场，大力发展肉鸭、肉鸡等规模养殖；加快发展百里核桃产业带，新增薄皮核桃种植面积9.7万亩；抓好精品蔬菜带建设，新发展高标准设施蔬菜5万亩。

　　三是提升产业化经营水平。大力培育产业化龙头，推进棉花、果品、蔬菜等农副产品深加工，启动特色高效农业示范园建设，加快农业产业化步伐。力争新增年销售收入5000万元以上农业产业化龙头企业9家，每个县(市、区)至少新建1个市级以上农业产业化项目。

　　四是加强农村基础设施建设。抓住国家和省加快水利改革发展的机遇，加快实施“两引一建”工程，抓好农田水利基本建设；推进县乡公路改造和农村公路建设养护，完成新一轮农村电网改造，促进农村通讯全面升级，切实抓好新民居示范村建设，改善农民生产生活条件。

　　五是拓展农民增收渠道。不折不扣落实各项强农惠农富农政策，大力发展农村二、三产业，鼓励发展观光农业、乡村旅游等，积极发展劳务经济，持续增加农民收入，努力使广大农民群众的日子过得更加殷实、生活变得更加美好！

　　（三）以转型升级为核心，提升工业经济发展水平。坚持走新型工业化道路，大力实施技术改造和创新，促进工业加快发展、加速转型、提质增效。

　　一是深入实施“千百工程”。落实好支持千亿产业和百亿企业发展的各项政策措施，通过新上一批产业支撑项目、延伸链条项目和现有企业提质扩能项目，壮大主导产业和骨干企业，促进一批成长型企业快速发展，力争新增一批销售收入超50亿元、100亿元的企业，装备制造、新能源、煤盐化工、新型建材四大产业增加值占全部工业的比重提高3.5个百分点。

　　二是改造提升传统产业。我市传统产业有较好的基础，在经济发展中仍具有重要支撑作用。我们要深入开展“对标行动”，加大技改投入力度，改进技术工艺、提升装备水平、提高加工深度，努力把我市的传统产业做大做强做优。突出抓好晶龙、中煤旭阳等20家转型升级重点企业，促进钢铁、纺织服装等传统产业和自行车、轴承等特色产业晋档升级。鼓励和引导企业抓住当前国际市场需求疲软、国家鼓励先进技术和产品进口的有利时机，积极购置技术专利、先进装备，提高产品的科技含量和附加值。

　　三是培育发展新兴产业。研究制定促进新兴产业发展的政策措施，大力引进高新技术企业、重大科研成果转化项目和行业领军人才，加快培育发展后续战略支撑产业，力争在新能源汽车、新材料等领域取得突破。

　　四是加快科技创新步伐。鼓励有条件的企业建立工程技术研究中心，重点支持旭阳煤化工等申报国家级工程技术研究中心，加快市开发区光伏新能源研究院建设。大力开展科技招商，推动产学研结合，加快建设产业技术创新联盟。支持申报高新技术企业和创新型试点企业，加大专利申报、引进力度，提高科研成果转化的数量和效率。引导企业大力研发新产品，开发省级新产品100项以上。加强产品质量管理，大力推进品牌建设，力争新增中国驰名商标3件。

　　五是强力推进节能减排。加快推进重点工程，抓好新老“双三十”单位的节能减排，坚决淘汰落后产能，大力发展循环经济，确保完成省下达的各项约束性指标。

　　六是加强工业运行调节。在当前形势下，支持现有企业发展，对于实现经济平稳较快增长，比招商引资更重要、更直接、更有效，必须落实好市县长联系企业、企业间产需对接、银企对接、校企对接等措施，充分发挥市域消费市场的作用，为企业提供订单支持；加强对重点企业煤电油气运的协调，及时帮助他们排忧解难。企业家是社会的宝贵财富，我们一定要倍加关心和支持企业家，使他们在事业上得到更多的保障、在社会上得到更多的尊重！

　　（四）以扩大规模、丰富业态为目标，加快振兴发展服务业。切实加大支持力度，加快发展新兴服务业，改造提升传统服务业，培育壮大龙头企业，力争服务业增加值增长11%左右。

　　一是做大做强金融业。增多做强金融主体，支持邢台银行、农村信用社等加快发展，力争组建2家农村商业银行，小额贷款公司实现县县全覆盖，河北银行、交通银行在我市设立分支机构。积极优化金融生态环境，创新丰富金融产品，努力扩大信贷投放。鼓励企业通过上市、债券等增加融资，重点支持旭阳化工等企业实现上市。

　　二是加快发展旅游业。落实好旅游业发展总体规划，积极引进战略投资者，开发建设百里太行旅游产业带；开展A级景区和乡村旅游示范点创建活动，鼓励申报4A级景区；加快推进大峡谷、岐山湖旅游休闲综合体等重点项目，完善道路交通等配套设施，提高旅游业发展水平。

　　三是改造提升商贸物流业。支持家乐园、好望角等龙头企业发展壮大，推进世贸天街、中北世纪城等商业综合体加快建设，开工建设凰家广场、邢台北国、守敬水城、红星美凯龙家具城等项目，力争培育一批销售收入超十亿元的服务业龙头企业，有条件的县（市、区）都要建设1个商业综合体项目，提升沙河玻璃、清河羊绒等一批特色专业市场。

　　同时，鼓励发展社会养老、社区服务等便民利民的服务业，做好农超对接等工作，提高城乡居民的消费水平。

　　（五）以上水平、蓄能量为导向，提速新型城镇化进程。继续推进城镇建设改造，拓展空间，完善功能，强化管理，提升品位，促进城镇建设上水平，力争城镇化率提高1.5个百分点。

　　一是深入推进“一城五星”统筹发展。加快“五星”公共基础设施、产业园区、快速路、绿廊等建设，进一步完善实施财政、税收等激励性政策措施，不断提高统筹发展水平。

　　二是集中力量加快新区建设步伐。邢台新区，充分发挥河北顺德投资集团融资主体作用，拓宽融资渠道，开工建设市博物馆、文化艺术中心、就业和社会保障服务中心、行政服务中心、城市展馆、会议中心等八大公建项目，完成六条主要道路建设。七里河新区，加大招商引资力度，抓好滨水商务休闲娱乐中心、百泉公园、健康“绿道”等重点工程建设，努力打造高品位的新亮点。高铁新区，开工建设高铁站综合客运枢纽、站前广场等项目，加快建设通往高铁站的五条主要道路，谋划启动商业金融、商贸物流等功能板块的开发建设。

　　三是强力推进重点工程建设。以园林城市创建为契机，加快实施市区城镇化建设百项重点工程。有序推进城中村、旧城区、危旧房和棚户区改造项目，加快推进“三园一圃”、“两围十街”、“四河两带”等重点绿化工程，扎实推进工业企业退市进园工作，谋划推进育英街和建设大街地道桥建设项目，加快辰光五星级酒店建设进度，晶龙五星级酒店尽早开工，促进市区已取得土地使用权的闲置地块加快建设。

　　四是强化规划引领和城市管理。进一步明确城市发展定位和功能分区，完善城市发展规划，强化规划刚性约束，努力打造具有独特风格和个性魅力的宜居城市。提升城市精细化管理水平，继续加大对违章建筑、不雅建筑、广告牌匾和环境卫生的整治力度，对主要街道、出市口、重点部位实施绿化、美化、净化、亮化，深入推进数字城市建设，完善智能化交通指挥系统。

　　五是完善县城镇功能。根据产业发展定位和要素承载容量，搞好县城镇功能规划设计，每年有计划地建设一批重点工程，提高承载能力。统筹搞好园区、城镇的规划和建设，启动建设县城之间的快速路，加快构建要素聚集、流动平台。认真做好省级园林县城创建工作。

　　六是加快重大基础设施建设。加快实施邢汾高速、邢黄高速、京港澳高速改扩建、邢黄铁路等“东出西联、南承北接”重大交通工程，邢和铁路力争年内开工，环城高速加快建设，启动石林高速立项和邢台机场民航复航征地拆迁及航站楼建设，加快南北大道建设步伐，积极推进任县热电联产等重大能源项目。城镇是生产要素的集中区，是现代生活方式的集合体，我们一定要使城镇功能更加完善、生活更加舒适、容貌更加亮丽！

　　（六）以改革开放为动力，增强经济发展活力。把改革作为破解发展难题的根本途径，把开放招商作为实现跨越发展的必由之路，不断向广度和深度拓展。

　　一是坚持不懈地扩大开放招商。抓住省深化与央企战略合作的有利时机，加强与新兴际华等大企业、大集团的对接，推动与中航、中盐、中建材等大集团合作协议的落实，深化与中钢、中煤等大集团的合作，力争实现重大项目突破。突出产业招商、企业招商，引导企业通过并购联合等形式，与战略投资者开展合作，支持骨干企业依托业务链、产业链吸引关联企业落户我市。完善招商机制，主要领导带头走出去登门招商，以情招商、以诚招商；切实为客商搞好服务，努力实现以商招商、组团引进；不断提升招商机构和人员的素质能力，积极开展专业招商、中介招商、网络招商。同时，努力扩大进出口，优化出口商品结构，提升出口规模和效益，邢台海关建成运行。

　　二是满腔热忱地支持民营经济发展。坚持非禁即准的原则，放手发展民营经济，深入推动全民创业，激发各类市场主体的创业热情，增多做强市场主体；建立健全融资担保、人才培训、技术支持等服务平台，加大对中小微型企业的扶持力度，鼓励有条件的民营企业上市融资，培育一批有较强市场竞争力的大型企业集团。实施企业家素质提升工程，推进民营企业管理创新、技术创新和装备更新，促进民营经济快速发展，力争增加值增长16%。

　　三是积极稳妥地推进重点领域改革。继续深化医药卫生体制、财税体制等改革；推进市属经营性国有资产监管全覆盖；稳步推进工商、质监行政管理体制调整和事业单位分类改革；健全和完善农村土地承包经营权流转机制，深化农村集体林权等改革。

　　（七）以打造科学发展平台为引领，培植可持续增长极。集合要素资源，强化载体支撑，大力培育带动产业聚集、持续发展的强劲引擎。

　　一是倾力打造科学发展大平台。以市开发区为核心，以东南板块（沙河、南和、任县）、西北板块（邢台县、内丘）和七里河板块为支撑，以市区周边省级开发区、工业聚集区为依托，采取“一区多园”模式，加强规划引导、强化政策支持、创新管理机制，大力发展战略新兴产业、特色主导产业、现代服务业等，培育打造有较强区域影响力的科学发展大平台，带动辐射全市经济快速发展。各县（市、区）也要积极谋划自己的科学发展平台，打造一批聚集效应强、发展速度快的经济增长极。

　　二是提升开发区、工业聚集区等各类园区发展水平。加大资金投入力度，加快路水电气讯等基础设施建设，市级以上园区全部达到“七通一平”，省级园区力争达到“九通一平”。进一步明确园区发展定位，严把项目入驻门槛，省、市重点项目必须进园区，园区内项目投资规模要实、强度要高，建设进度要快。理顺管理体制，建立和完善研发、信息等公共服务平台，增强园区发展活力。市开发区、清河开发区要积极申报国家级开发区，19家省级开发区、工业聚集区要确保考核达标，同时力争有更多市级园区跨入省级序列。

　　三是努力壮大县域经济。以黑龙港流域为重点，抓住省加大扶持力度的难得机遇，谋划整体发展思路，加快县域经济发展步伐。加大引导力度，促进项目向园区集中，园区向县城集中，人口向城镇集中，做大做强县城经济。在积极抓好大项目、培育壮大特色产业的同时，大力引进和支持符合产业政策的小项目，做大企业群体。扶持中小企业加快发展，充分发挥融资担保中心作用，加大技术人才引进、技术工人培训力度，帮助中小企业解决融资、用工等难题。力争到“十二五”末，排位全省“三十弱”的县经济发展综合实力大幅跃升，跨入前100位行列；百位以内的县提升位次，力争有更多县(市)跨入前“三十强”！

　　（八）以优化发展环境为保障，营造聚集要素的洼地。牢固树立环境是核心竞争力的理念，建立完善长效机制，动真招、出重拳，全力打造一流发展环境。

　　一是营造务实高效的政务环境。深化行政审批制度改革，大力削减审批、收费事项，努力打造“零收费、零罚款”城市。提升行政服务中心的服务水平，实施并联审批、项目代办等制度，优化服务流程，提高办事效率。加强效能检查，畅通投诉渠道，下大力解决以部门中层为重点的“中梗阻”现象。狠抓反面典型，坚决杜绝吃拿卡要、乱检查、乱收费等行为。

　　二是营造公平公正的商务环境。大力倡导公正执法、文明执法，有效整顿和规范市场经济秩序，依法保护投资者合法权益。加大企业周边治安环境的整治力度，严厉打击敲诈勒索、无理刁难、阻碍施工等不法行为。

　　三是营造亲商安商的社会环境。推进诚信体系建设，强化社会诚信意识，认真兑现对投资者的承诺，解决好客商就医、子女就学等实际问题，以良好的环境吸引客商、留住客商，使邢台真正成为客商投资的热土、兴业的家园！

　　各位代表，加快发展、跨越赶超是时代的要求、人民的期盼，只要我们万众一心，团结奋斗，标准向先进看齐，精力向任务聚焦，就一定能把邢台的事业推上一个新的发展高度！

　　四、强力推进以改善民生为重点的社会建设

　　把实现好、维护好、发展好广大人民群众的根本利益，作为我们一切工作的出发点和落脚点，让全市人民共享经济发展成果，共同维护和谐稳定大局。

　　（一）优先发展教育人才事业。大力实施教育振兴工程，改善办学条件，加强校长和教师队伍建设，推进教育教学创新，整治校园周边环境，加强中小学校车管理，巩固提高高中阶段教育普及水平，全面提升教育教学质量；推动职业教育集团化发展，中职学校规范化建设，加快市区和皇寺两个职教园区建设；加强高等院校特色学科、重点学科建设，全日制普通高校本科生生均公用经费一步提高到12000元。完善高层次人才引进、使用机制，实施“百千万”人才引进工程，努力打造一批优秀科研创新团队；实施青年科技人才资助计划，支持高层次人才承担重大项目；发挥邻近京津优势，吸引一批“星期天工程师”、“星期天院士”来邢服务。

　　（二）提高医疗卫生事业发展水平。完善基层医疗卫生服务网络，加强县乡村和社区医疗卫生机构建设，抓好基层卫生人才队伍建设，进一步提高基本公共卫生服务均等化管理和服务水平。大力支持中医药事业发展，改善中医医疗机构基础设施。做好人口计生工作，综合治理人口性别比问题，稳定低生育水平，提高人口素质。积极发展竞技体育，承办好河北省第十七届大学生运动会，加快群众健身设施建设，广泛开展全民健身活动。

　　（三）推动文化事业大发展。全面落实党的十七届六中全会精神，大力加强社会主义核心价值体系建设，深入开展群众性精神文明创建活动；加大对文化事业、文化产业的投入，完善文化服务体系；进一步深化专业院团改革，激活文艺演出市场；加快发展文化产业，鼓励和支持工笔画、老粗布、沙画、艺术玻璃等产业化发展；进一步加强历史文化名城的规划与建设，打造一批具有历史文化特色的商业街区；加强文物和非物质文化遗产保护，深入挖掘文化内涵，支持文化精品创作，打造文化品牌。

　　（四）维护社会和谐稳定。加强和创新社会管理，协调推进城乡社区建设，提高社区自治和服务功能。加强信访调解，畅通群众利益诉求表达渠道。深入推进“六五”普法，加强法制宣传教育。强化社会治安综合治理，继续严厉打击涉黑涉恶、“两抢一盗”等各类违法犯罪行为。开展食品药品安全专项整治，确保人民群众饮食用药安全。认真抓好安全生产和消防、交通安全，坚决遏制重特大事故发生。健全公共安全应急体制，提高应急处置能力。抓好“菜篮子”工程，加强市场监管，保证人民群众生活必需品供应。有效保护妇女儿童合法权益，积极发展老龄、残疾人和慈善事业。继续做好对口援建新疆等工作。推进双拥共建、民族宗教、外事侨务、防震减灾、人防气象、统计、档案、地方志等各项工作取得新进步。

　　各位代表，着眼于解决关系人民群众切身利益的突出问题，今年我们将集中力量实施十大民生工程：

　　一是就业促进工程。实施积极的就业政策，城镇新增就业5.4万人，应届高校毕业生就业率达到85%以上，转移农村劳动力21万人（次），确保城镇零就业家庭保持动态为零。

　　二是社保扩面提标工程。城乡居民养老保险制度实现全覆盖，医疗、生育保险实现市级统筹，企业退休人员基本养老金和城乡低保标准均提高10%，城镇居民医保和新农合补助标准提高到每人每年240元。

　　三是百姓安居工程。开工建设保障性安居工程住房22510套，确保建设进度、确保工程质量、确保分配公平。

　　四是困难群众救助工程。实施好城镇困难家庭综合救助措施，保障城镇困难群众的基本生活水平不因物价上涨而降低，并在就业、就医、就学等方面及时开展救助；关心农村特困群众的生活，加大“五保”供养工作力度。

　　五是农村饮水安全工程。解决25万农村人口的饮水不安全问题。

　　六是扶贫开发工程。以国家、省加大扶贫投入为契机，以黑龙港流域和太行山区为重点，全面启动“十二五”387个村的扶贫计划，实现3万贫困人口稳定脱贫。

　　七是学校医院提升工程。市区新建或改扩建幼儿园4所、中小学19所，其中标准化幼儿园2所、中小学7所，每个县（市）新建或改扩建标准化中小学4所，基本完成中小学校舍安全工程规划任务。加快市人民医院、眼科医院建设步伐，启动三院、二院、五院和中医院迁扩建项目，筹建市儿童医院；加快推进9家县级医院改扩建，每个县（市、区）都有1家二级甲等医院，提升乡镇卫生院服务能力。

　　八是文化惠民工程。市图书馆免费向社会开放，开工建设市博物馆、文化艺术中心等公益文化设施，完善提升乡镇综合文化站20个，农家书屋实现村村全覆盖。

　　九是便民服务工程。市区启动32条小街巷的综合整治，打通9条断头路，新建13个社区卫生服务站、22个治安岗亭、3个便民菜市场；新增“万村千乡市场工程”农家店300个，行政村覆盖率达到78%以上，加快697个空白村卫生室建设。十是生态环境改善工程。深入开展市区大气污染综合整治和农村环境卫生集中整治，对市区周边违规生产或污染环境的小板材、小石子、小石灰等企业，全部进行治理、搬迁或关停，拔掉“黑烟囱”27根；抓好太行山、丘陵区、环市区和主干道两侧的绿化；加快实施水生态修复工程和“一城五星”水系、市区风景水系规划，关停市区自备井57眼，谋划启动白马河综合治理项目，使邢台早日再现“泉城”风采，成为山青水秀的绿色之城、生态之城！

　　各位代表，实现共同富裕是我们的崇高目标，保障和改善民生是我们的神圣职责，我们一定要把工作重点进一步向民生倾斜，财力物力进一步向民生投放，让全市人民生活得更加幸福安康！

　　五、努力建设服务型、效能型政府

　　新形势、新任务，对政府工作提出了新要求，我们将本着对人民高度负责的态度，切实加强政府自身建设，不断提高行政效能和工作水平，更好地为发展服务、为基层服务、为群众服务。

　　（一）解放思想，激发创新活力。积极走出去学习经验、开阔眼界，坚决破除封闭保守的落后观念和因循守旧的惯性思维，打破条条框框的束缚，以创新的思维、改革的办法推动工作、促进发展。深入开展创先争优活动，牢固树立开拓意识、担当意识、争先意识，始终保持自信自强、奋发有为的志气，始终保持攻坚克难、志在必得的锐气，始终保持干事创业、争先进位的激情,努力创造无愧于历史、无愧于时代、无愧于人民的工作业绩。

　　（二）转变职能，增强服务能力。进一步强化公共服务和社会管理职能，把属于市场的职能还给市场，把属于政府的职能认真履行好。推进政府管理创新，加强电子政务建设，优质高效地为企业、为客商搞好服务。时刻牢记为人民服务的宗旨，把人民群众的需要放在首位，真心实意地为群众办实事、解难事，真正使发展成果惠及广大人民群众。

　　（三）改进作风，提升行政效能。扎实开展“效能提升年”活动，大力倡导说了就办、定了就干、干就干好的工作作风。强化执行，对重点工作、目标任务和重要事项，逐项明确责任分工，细化进度要求，挂图作战，跟踪问效，确保落实。真抓实干，察实情、办实事、求实效，决不能空喊口号、虚报浮夸、贻误发展。严明奖惩，建立和完善激励约束机制，强化责任追究，坚决杜绝不作为、乱作为、拖沓散漫、效率低下等行为，确保令行禁止、政令畅通，形成崇尚实干、跨越争先的浓厚氛围。

　　（四）依法行政，自觉接受监督。健全科学民主决策机制，加强行政决策风险评估，提高决策科学化水平。严格依照法定权限和程序履行职责，进一步规范行政行为，切实做到依法行政，提高政府公信力。认真执行人大及其常委会的决议、决定，依法接受人大及其常委会的法律监督，主动接受政协的民主监督，认真办理人大代表建议和政协提案，充分听取各民主党派、工商联、无党派人士、人民团体和人民群众的意见。

　　（五）从严治政，做到清廉为民。牢记两个“务必”，大力倡导艰苦奋斗、勤俭节约的风尚，严控“三公”经费支出，坚决制止铺张浪费和奢靡之风。坚持用制度管权、管人、管事，积极推进政务公开，让权力在阳光下运行。把清正廉洁作为第一操守，认真落实党风廉政建设责任制，严格执行廉洁从政各项规定，严肃查处各类违法违纪案件，努力建设人民满意、人民信赖的政府！

　　各位代表，邢台正处于经济社会加快发展的关键时期，全市人民信心百倍、豪情满怀，正朝着奋力走向振兴的宏伟目标阔步前进。让我们更加紧密地团结在以胡锦涛同志为总书记的党中央周围，在中共邢台市委的领导下，紧紧依靠全市人民，深入贯彻落实科学发展观，同心同德，迎难而上，锐意进取，奋力开拓，全面完成今年各项目标任务，推进经济社会实现更好更快更大发展，加快科学发展、富民强市进程，以优异成绩迎接党的十八大胜利召开！
